# Admissions for personality disorders in Italy from 1988 to 1998

**DOI:** 10.1186/1745-0179-2-20

**Published:** 2006-08-30

**Authors:** Giuseppe Guaiana, Corrado Barbui

**Affiliations:** 1 Clinica Psichiatrica Cantonale, Organizzazione Sociopsichiatrica Cantonale, Via Maspoli, Mendrisio, Canton Ticino, Switzerland; 2 Section of Psychiatry and Clinical Psychology, Department of Medicine and Public Health, University of Verona, Verona, Italy

## Abstract

**Background:**

Personality disorders affect a substantial proportion of the population. It is unclear, however, whether the burden of personality disorders on modern mental health services has been increasing. To fill this gap, we analyzed trends in admissions for personality disorders in Italy from 1988 to 1998.

**Methods:**

We used the yearly data from the Italian Central Institute of Statistics to analyse trends in the total number of admissions for personality disorders and in the total number of first admissions for personality disorders.

**Results:**

The absolute number of admissions for personality disorders almost trebled from 1988 to 1998, as well as the proportion of all psychiatric admissions that were for personality disorders. Whilst there has been a marked increase in the absolute number of first admissions, the proportion of all first psychiatric admissions that were for personality disorders showed a steady but modest increase, from 5.7% to 7.6%.

**Conclusion:**

In Italy, the burden of personality disorders on modern mental health services has been increasing. In terms of public health, these findings highlight the urgent need of developing policies to tackle the increasing demand of care of this difficult-to-treat patient population.

## Background

Personality Disorders (PDs) affect a substantial proportion of the population. While community surveys estimated that between 4.4 and 13% of individuals have a PD [1], surveys carried out in outpatient psychiatric settings estimated that nearly one third of psychiatric patients were diagnosed with one specific PD, and up to fifty percent received a general diagnosis of PDs [2]. Despite these figures, it remains unclear whether the burden of PDs on modern mental health services has been increasing. In North West Wales, a survey showed that PDs accounted for 9% of total admissions for psychiatric problems in 1996, while no PD patients were admitted 100 years ago [3]. However, hospital admissions for PDs have never been examined during a time frame of substantial stability in terms of bed availability and conceptualization of PDs. To fill this gap, we analyzed trends in admissions for PDs in Italy from 1988 to 1998.

## Methods

In Italy, the Italian Central Institute of Statistics (ISTAT) routinely collects data from health authorities on hospital admissions. These data are published in the health-care statistics yearbooks for the corresponding period. Data collection is based on the ICD-9-CM classification system. These figures were used to establish the total number of admissions for PDs and the proportion of all psychiatric admissions that were for PDs between 1988 and 1998. We additionally established the total number of first admissions for PDs and the proportion of all first psychiatric admissions that were for PDs. While the ISTAT system of data collection has always remained the same during the time frame considered in this analysis, a new system was implemented after 1998, and admission numbers were not recorded anymore. Previous analyses showed that ISTAT data have been shown to be useful for monitoring compulsory admissions [4], suicide rates [5] and other public health indicators [6].

## Results

**Figure 1:**
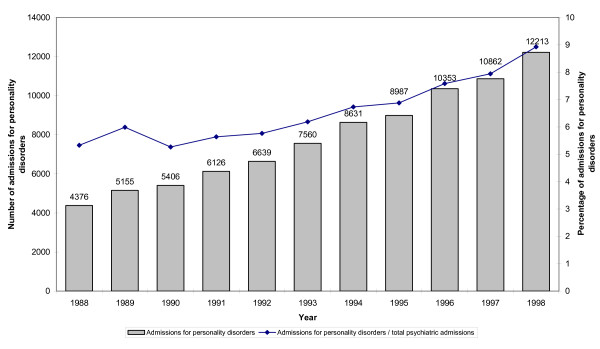
**Admissions for PDs**. Number of admissions for personality disorders in Italy from 1988 to 1998, and proportion of all psychiatric admissions that were for personality disorders.

The total number of admissions for PDs almost trebled from 1988 to 1998; similarly, the proportion of all psychiatric admissions that were for PDs progressively increased, from 5.3% to 8.9% (Figure 1).

**Figure 2:**
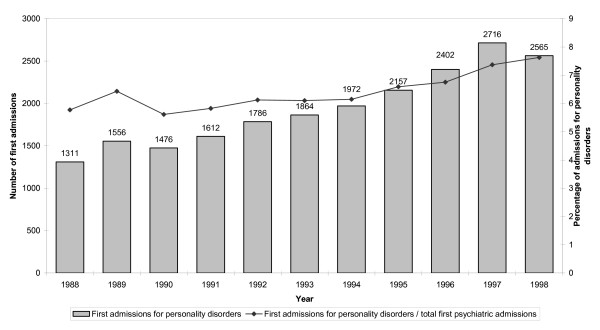
**First admissions for PDs**. Number of first admissions for personality disorders in Italy from 1988 to 1998, and proportion of all first psychiatric admissions that were for personality disorders.

The analysis of first admissions for PDs revealed that whilst there has been a marked increase in the absolute number of first admissions, the proportion of all first psychiatric admissions that were for PDs showed a steady but modest increase, from 5.7% to 7.6% (Figure 2).

## Discussion

In Italy, the burden of PDs on modern mental health services has been increasing. In 1998, 9% of all psychiatric admissions were for PDs.

A limitation of this analysis is the possible low quality of reporting by hospitals. Only clinical diagnoses of PDs are routinely collected, and no data are available to check the degree of coherence between this diagnostic information and research diagnoses of PDs. Given the difficulties of obtaining reliable diagnoses of some PDs [7], it is possible that the burden of PDs has been underestimated. In addition, the system work in such a way that no data are available on specific PDs, and therefore it remains unclear whether these increasing trends are mostly explained by the increasing burden of a specific PD, for example borderline personality disorder. Although we acknowledge these limitations, during the index years considered in this analysis the system of data collection has never changed. Likely, therefore, the possibility of low quality of reporting should not have affected the analysis of trends over the years. Another limitation is that we have no reliable data to establish whether these trends represent a genuine increase in hospital admissions or a greater awareness of the diagnosis. However, given that no major changes in the conceptualization of PDs has occurred during the time frame considered in this analysis, it seems that these trends cannot be entirely attributed to a greater awareness of the illness.

## Conclusion

In terms of public health, these findings highlight the urgent need of developing policies to tackle the increasing demand of care of PD patients. Although to many clinicians PD remains a specialist subject, in many countries the possibility of developing specific facilities, in the public sector, seems unrealistic. Conversely, modern mental health services could probably develop specific skills for the psychological management of these patients, recognising that is almost impossible to be engaged in clinical psychiatry without coming across the problem of PDs.

## Abbreviations

PD = Personality Disorder

PDs = Personality Disorders

## Competing interests

The author(s) declare that they have no competing interest.

## Authors' contributions

CB conceived the study. GG implemented the idea, did the literature search and wrote the first draft of the article, mainly the Background, Methods and Results section. CB supervised the writing process and added the Discussion and Conclusion section.
